# A Study of the Use and Outcomes From Respiratory Viral Testing at a Mid-Sized Children’s Hospital

**DOI:** 10.1177/0009922818809523

**Published:** 2018-10-26

**Authors:** Chelsea Zhu, Sabeen Sidiki, Brittany Grider, Brian Fink, Nicole Hubbard, Deepa Mukundan

**Affiliations:** 1 University of Toledo, Toledo, OH, USA; 2 ProMedica Toledo Hospital, Toledo, OH, USA

**Keywords:** antibiotic use, respiratory viral panel, length of stay, antibiotic stewardship

## Abstract

This study was a retrospective analysis of inpatient and emergency department (ED) data on respiratory pathogen panel (RPP) testing between December 16, 2013, and December 15, 2015, at a mid-sized children’s hospital. We assessed whether RPP decreases antibiotic days of therapy and length of hospital stay for pediatric patients with acute respiratory infections. In the inpatient population, patients testing positive with RPP were given fewer antibiotic days of therapy (2.99 vs 4.30 days; *P* = .032) and had shorter hospital stays (2.84 vs 3.80 days; *P* = .055) than patients testing negative. In the ED population, patients testing positive with RPP received fewer discharge prescriptions for antibiotics than patients not tested (8.8% vs 41.1%; *P* < .001). RPP use was more prevalent in admitted patients than in ED patients (78.9% vs 7.3%; *P* < .001). Our results suggest that RPP testing curbs antibiotic use and decreases length of hospital stay.

## Introduction

Acute respiratory infection (ARI) is a leading cause of hospitalization for children in developed countries.^1^ In hospitalized children younger than 5 years of age, 66% of ARI cases are caused by viral infections.^2,3^ Antimicrobial agents are prescribed almost twice as often as necessary, resulting in an estimated 11.4 million unnecessary antibiotic prescriptions per year.^4^ Such antimicrobial prescription practices drive increased and accelerated antibiotic resistance of bacteria, drug-related adverse effects, and unnecessary medical costs.^5^ To address these issues, Infectious Diseases Society of America, the Society for Healthcare Epidemiology of America, and the Pediatric Infectious Diseases Society strongly recommend implementation of evidence-based antibiotic stewardship programs.^6^ Newer tests for detecting viral infections, such as respiratory pathogen panels (RPPs) have been introduced for more judicious therapy in the clinical setting.^7^

The RPP was introduced to ProMedica Toledo Children’s Hospital on December 16, 2014, and used to test nasopharyngeal samples by polymerase chain reaction (PCR) evidence of numerous respiratory viruses and bacteria. We hypothesized that the introduction of RPP would result in the following:

1. Decrease antibiotic days of therapy (DOT) for pediatric inpatients admitted for ARI
2. Decrease length of stay in the hospital for pediatric inpatients admitted for ARI
3. Decrease number of ED pediatric patients with ARI who receive antibiotic prescriptions on discharge

## Materials and Methods

### Setting

**Figure 1.**
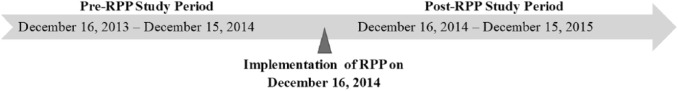
Timeline of retrospective analysis of inpatient unit and emergency department at ProMedica Toledo Children’s Hospital. RPP, respiratory pathogen panel.

This was an institutional review board–approved retrospective analysis of pediatric patient data collected between December 16, 2013, and December 15, 2015, at ProMedica Toledo Children’s Hospital (Figure 1). Because the RPP was first implemented on December 16, 2014, the 2 study periods were defined as follows: pre-RPP (December 16, 2013, to December 15, 2014) and post-RPP (December 16, 2014, to December 15, 2015).

### Inclusion Criteria

Pediatric patients from 1 month to 18 years of age with uncomplicated acute respiratory tract infections admitted into the hospital or seen in the ED were included for analysis. Those seen in the ED also had to be discharged from the ED to be included. The patients involved in the study were determined by probing the ProMedica Toledo Children’s Hospital patient diagnoses list with the International Classification of Diseases (ICD) Ninth Revision and 10th Revision codes for bronchiolitis, pneumonia, lower respiratory tract infections, and upper respiratory tract infections (ICD codes available on request).

### Exclusion Criteria

Patients with uncomplicated respiratory tract infections were defined as those without the following: chronic respiratory diseases, other chronic medical illnesses, complications from the respiratory tract infection, nonrespiratory infections, and admission to the pediatric intensive care unit for longer than 24 hours.

### Data Collection

**Figure 2.**
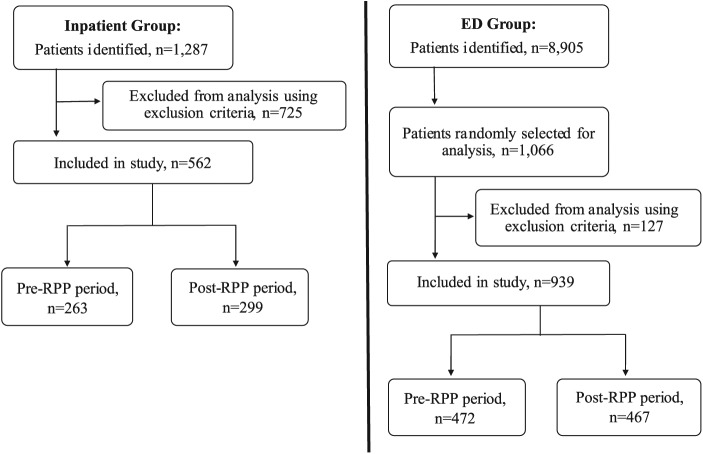
Selection process and inclusion criteria for hospital inpatients and emergency department (ED) patients in this study. RPP, respiratory pathogen panel.

Five hundred and sixty-two pediatric inpatients and 939 pediatric ED patients were ultimately included in the study (Figure 2). Review of electronic medical records was performed for every time period of the study. The data abstracted from the electronic medical record included demographics, results from radiology and microbiology laboratories, inpatient antibiotic therapy, and discharge prescriptions.

### RPP Testing

Samples for RPP testing were collected via nasopharyngeal swabs. RPP was performed through PCR detection by BioFire FilmArray Assay, which identifies common viral pathogens (respiratory syncytial virus, influenza, parainfluenza, rhinovirus/enterovirus, adenovirus, coronavirus, and human metapneumovirus), as well as common bacterial pathogens (*Mycoplasma pneumoniae, Chlamydia pneumoniae*, and *Bordetella pertussis*).

### Statistical Analysis

For the inpatient population, our initial probe with ICD codes returned 598 patients from the pre-RPP period and 689 patients from the post-RPP period (Figure 2). Two hundred and sixty-three patients from the pre-RPP period and 299 patients from the post-RPP period met inclusion criteria. For the ED patient population, 8905 ED patients were identified by ICD codes: 4556 patients in the pre-RPP period and 4349 patients in the post-RPP period. The power necessary to produce a confidence interval of 95% and margin of error of 5% was 377 each in the pre- and post-RPP periods. To generate a sample population of at least 377 pre-RPP and 377 post-RPP patients, 533 pre-RPP patients and 533 post-RPP patients were randomly selected using a computer algorithm due to an expected 88% inclusion rate. The total ED sample population reflected the true proportions of patients seen each month in the ED. Four hundred and seventy-two patients from the pre-RPP period and 467 patients from the post-RPP period met inclusion criteria. Per Infectious Diseases Society of America guidelines, the rates of antimicrobial use were assessed using the DOT method.^6^ Data were analyzed with IBM SPSS Statistics, Version 24.

## Results

### Inpatient Group

**Figure 3.**
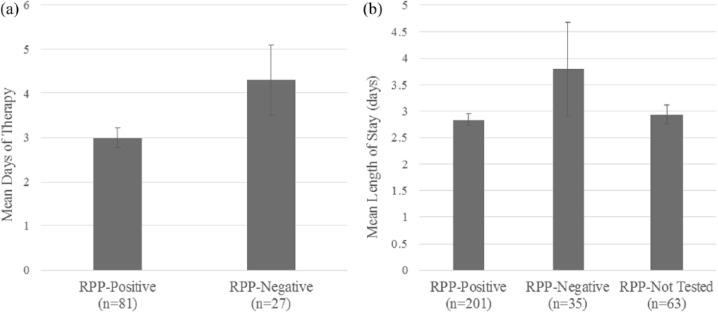
Inpatient post-respiratory pathogen panel (RPP) study period for acute uncomplicated respiratory tract infection. (a) Patients who tested RPP-positive had fewer antibiotic days of therapy compared with RPP-negative patients (*P* = .032). (b) RPP-positive patients had shorter lengths of stay at the hospital compared with RPP-negative patients (*P* = .055).

When looking at patients with acute uncomplicated respiratory tract infections in the post-RPP study period, patients testing positive with RPP (RPP-P) received fewer antibiotic DOT than patients testing negative with RPP (RPP-N) in the inpatient unit (2.99 vs 4.30 mean DOT; *P* = .032; 95% confidence interval = −2.503 to −0.115; Figure 3a). In addition, using multiple comparison analysis of ANOVA (analysis of variance) results between RPP-P, RPP-N, and patients who were not tested with RPP (RPP-NT), RPP-P patients had a trend toward shorter mean length of stay at the hospital when compared with RPP-N patients (2.84 vs 3.80 days; *P* = .055; 95% confidence interval = −1.935 to 0.017; Figure 3b). RPP-NT patients had a mean length of stay of 2.94 days. No differences were found in comparison with RPP-NT patients.

When looking at specific antibiotic clusters (azithromycin, third-generation cephalosporins, and aminopenicillins), no statistically significant differences were found in mean DOT for any antibiotic group between pre- and post-RPP inpatient study periods. Furthermore, RPP-N patients were older than RPP-P patients and RPP-NT patients with ANOVA (mean 6.21 vs 2.40 and 2.43 years; *P* = .008).

### ED Group

**Figure 4.**
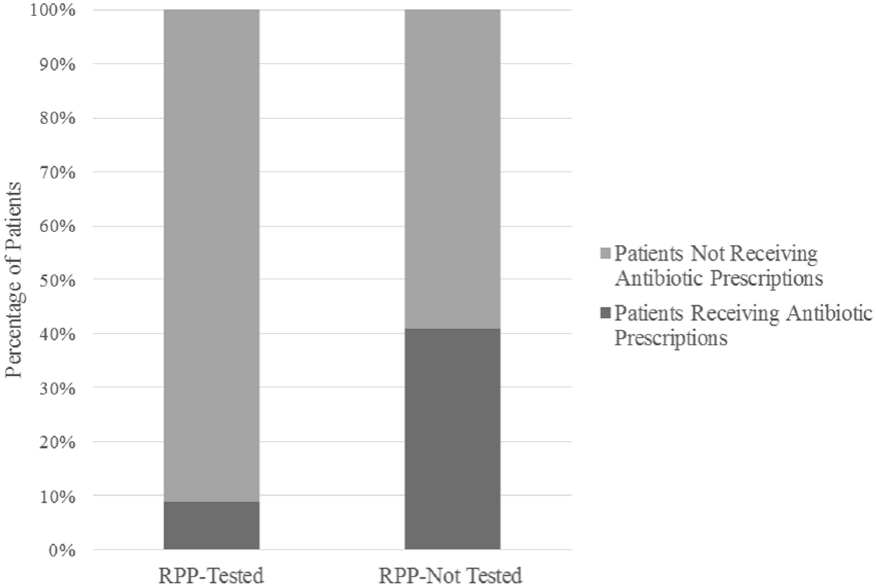
Emergency department patient post-RPP study period for acute uncomplicated respiratory tract infection. Fewer patients received antibiotic prescriptions on discharge when tested with respiratory pathogen panel (RPP) compared with those not tested with RPP (χ^2^ = 13.57; *P* < .001).

In the post-RPP study period for ED patients, 3 out of 34 RPP-P patients and 178 out of 433 RPP-NT patients were prescribed antibiotics on discharge. Fewer RPP-P patients were prescribed antibiotics on discharge when compared with RPP-NT patients (8.8% vs 41.1%; χ^2^ = 13.57; *P* < .001; Figure 4). There was no statistically significant difference in the number of patients who received antibiotics on discharge from ED between the pre- and post-RPP study periods.

### Inpatients Versus ED Patients

**Figure 5.**
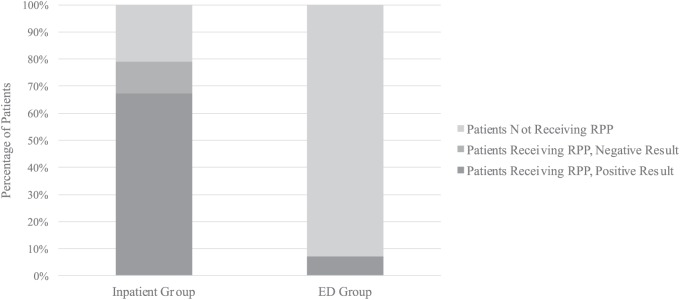
Inpatient versus emergency department (ED) post-RPP study period for acute uncomplicated respiratory tract infection. Respiratory pathogen panel (RPP) usage was more prevalent in the pediatric inpatient unit when compared with the ED (χ^2^ = 408.56; *P* < .001). All ED patients receiving RPP had a positive test result (n = 34).

Of the 299 patients in the inpatient setting during the post-RPP study period, there were 63 RPP-NT patients (21.1%), 201 RPP-P patients (67.2%), and 35 RPP-N patients (11.7%). Of the 467 patients in the ED during the post-RPP period, there were 433 RPP-NT patients (92.7%) and 34 RPP-P patients (7.3%) and all ED patients who were tested with RPP received a positive result. RPP usage was more prevalent in the pediatric inpatient unit than the ED (78.9% vs 7.3%; χ^2^ = 408.56; *P* < .001; Figure 5).

## Discussion

### Inpatient Group

Our analysis of pediatric inpatients with acute uncomplicated respiratory tract infection revealed that a positive RPP test correlates with a decrease in antibiotic DOT. Previous studies^7,8^ also show a decrease in antibiotic use in pediatric patients who test positive for viral pathogens during peak prevalence of respiratory viral infections. However, these studies analyzed the use of viral-only pathogen panels that could not detect concomitant bacterial and viral respiratory infections. The RPP test in our study detects certain pathogens both bacterial and viral in nature. This may alleviate some concern of missing a bacterial infection in light of a positive viral pathogen test, which could aid clinical judgment for antibiotic management.

A trend toward decreasing length of stay when compared with patients testing negative with RPP was noted in our study. We excluded patients with chronic illnesses and severe respiratory problems in our study. A previous study^8^ determined that positive respiratory viral testing corresponds with decreased hospital length of stay in select pediatric inpatient populations who had complicated medical history. Thus, the utility of RPP testing for decreasing length of stay may not necessarily depend on the patient’s underlying clinical picture as demonstrated in our analysis.

### ED Group

Our study determined a statistically significant decrease in antibiotic prescriptions for patients tested with RPP in the ED when compared with those who were not tested at all. Our study was limited by the relatively small number of ED patients who were tested with RPP; all patients who were tested were found to be positive for viral respiratory pathogens. To our knowledge, there is limited evidence on the impact of RPP testing in the ED for management of acute uncomplicated pediatric respiratory infections.

We believe that barriers to RPP testing in ED patients should be identified, so that RPP testing can enhance the clinical judgment required for the judicious use of antibiotics. The cost of RPP is likely the biggest deterrent for effective ED use. In our facility, the cost varies, and insurance coverage is almost nonexistent for the test, so families may have to pay out of pocket for the test. A recent study^9^ discovered that point-of-care viral PCR testing in pediatric ED patients has the potential to curb inappropriate use of antibiotics, reduce the cost of patient hospital visits, and decrease time spent in the ED. These benefits can occur only if the cost of RPP is manageable, and its use will help decrease antibiotic use in the ED.

## Conclusion

The introduction of RPP on December 16, 2014, at ProMedica Toledo Children’s Hospital, a mid-sized children’s hospital, resulted in a decrease in antibiotic DOT for pediatric inpatients, a trend in decreasing length of stay for pediatric inpatients, and a decrease in antibiotic prescriptions on discharge for ED patients. We believe that respiratory viral testing is effective for helping guide clinical judgment regarding antibiotic use and also the duration of hospitalization. Because inpatient RPP use is significantly more prevalent than ED use, future research should identify current barriers to administering RPP tests in the ED, one of which is likely the cost of the test.

## Author Contributions

CZ: Made a substantial contribution to the concept, design of the work, data acquisition, analysis, interpretation of data, funding acquistion and drafted the article. SS: Made a substantial contribution to the design of the work; data acquisition, analysis, interpretation of data, and first draft of the manuscript. BG: Made a substantial contribution to the concept and design of the work; data acquisition, analysis and interpretation of data, and revised it critically for important intellectual content. BF: Data analysis and statistical interpretation. NH: Concept, design and data inerpretation DM: Made a substantial contribution to the concept, design of the work; and interpretation of data. Reviewed and revised the manuscript critically for important intellectual content and approved the version to be published.
